# Economic Burden of Postoperative Neurocognitive Disorders Among US Medicare Patients

**DOI:** 10.1001/jamanetworkopen.2020.8931

**Published:** 2020-07-31

**Authors:** M. Dustin Boone, Brian Sites, Friedrich M. von Recklinghausen, Ariel Mueller, Andreas H. Taenzer, Shahzad Shaefi

**Affiliations:** 1 Department of Anesthesiology, Dartmouth-Hitchcock Medical Center, Lebanon, New Hampshire; 2 Department of Anesthesia, Harvard Medical School, Critical Care and Pain Medicine, Massachusetts General Hospital, Boston, Massachusetts; 3 The Dartmouth Institute for Health Policy and Clinical Practice, Lebanon, New Hampshire; 4 Department of Anesthesia, Harvard Medical School, Critical Care and Pain Medicine, Beth Israel Deaconess Medical Center, Boston, Massachusetts

## Abstract

**Importance:**

Postoperative neurocognitive disorders (PNDs) after surgical procedures are common and may be associated with increased health care expenditures.

**Objective:**

To quantify the economic burden associated with a PND diagnosis in 1 year following surgical treatment among older patients in the United States.

**Design, Setting, and Participants:**

This retrospective cohort study used claims data from the Bundled Payments for Care Improvement Advanced Model from 4285 hospitals that submitted Medicare Fee-for-service (FFS) claims between January 2013 and December 2016. All Medicare patients aged 65 years or older who underwent an inpatient hospital admission associated with a surgical procedure, did not experience a PND before index admission, and were not undergoing dialysis or concurrently enrolled in Medicaid were included. Data were analyzed from October 2019 and May 2020.

**Exposures:**

PND, defined as an *International Classification of Diseases, Ninth* or *Tenth Revision*, diagnosis of delirium, mild cognitive impairment, or dementia within 1 year of discharge from the index surgical admission.

**Main Outcomes and Measures:**

The primary outcome was total inflation-adjusted Medicare postacute care payments within 1 year after the index surgical procedure.

**Results:**

A total of 2 380 473 patients (mean [SD] age, 75.36 (7.31) years; 1 336 736 [56.1%] women) who underwent surgical procedures were included, of whom 44 974 patients (1.9%) were diagnosed with a PND. Among all patients, most were White (2 142 157 patients [90.0%]), presenting for orthopedic surgery (1 523 782 patients [64.0%]) in urban medical centers (2 179 893 patients [91.6%]) that were private nonprofits (1 798 749 patients [75.6%]). Patients with a PND, compared with those without a PND, experienced a significantly longer hospital length of stay (mean [SD], 5.91 [6.01] days vs 4.29 [4.18] days; *P* < .001), were less likely to be discharged home (9947 patients [22.1%] vs 914 925 patients [39.2%]; *P* < .001), and had a higher incidence of mortality at 1 year after treatment (4580 patients [10.2%] vs 103 767 patients [4.4%]; *P* < .001). After adjusting for patient and hospital characteristics, the presence of a PND within 1 year of the index procedure was associated with an increase of $17 275 (95% CI, $17 058-$17 491) in cost in the 1-year postadmission period (*P* < .001).

**Conclusions and Relevance:**

The findings of this cohort study suggest that among older Medicare patients undergoing surgical treatment, a diagnosis of a PND was associated with an increase in health care costs for up to 1 year following the surgical procedure. Given the magnitude of this cost burden, PNDs represent an appealing target for risk mitigation and improvement in value-based health care.

## Key Points

**Question:**

What is the association of patients who are diagnosed with a postoperative neurocognitive disorder with total Medicare health care expenditures in the year after their surgical procedure?

**Findings:**

In this retrospective cohort study including nearly 2.4 million Medicare patients, after adjusting for patient and hospital characteristics, the presence of a postoperative neurocognitive disorder was associated with an increase of $17 275 in payments in the 1 year after the index surgical procedure.

**Meaning:**

These findings suggest that in older patients who underwent surgical treatment, a diagnosis of a postoperative neurocognitive disorder was associated with an increase in health care costs for up to 1 year after treatment, emphasizing the economic burden associated with this potentially modifiable complication.

## Teaser

This cohort study examined the association of postoperative neurocognitive disorders with health care expenditures among US Medicare patients.

## Introduction

Cognitive impairment is the most common complication experienced by older adults after surgical treatment.^1^ The incidence of cognitive impairment ranges from 10% to 65% and varies depending on a variety of factors, such as age, level of education, sex, comorbidities, surgery type, and assessment methods.^2,3^ Although delirium is often most apparent in the hospital setting and has been rigorously studied in the immediate postoperative phase, other distinct yet potentially connected forms of postoperative cognitive dysfunction continue through, and often past, discharge.^4,5,6,7^ Recently, an expert panel proposed the term *perioperative neurocognitive disorders* (PND) to realign the field with allied specialty nomenclature and presumptive pathophysiological characteristics.^8^ A diagnosis of PND describes the different types of cognitive disorders encountered in the perioperative setting from immediately postoperatively out to 1 year.^9^ Based on the diagnostic criteria from the *Diagnostic and Statistical Manual of Mental Disorders* (Fifth Edition) (*DSM-5*),^10^ PND definitions are congruent with coding for postoperative delirium, delayed neurocognitive recovery, and mild or major neurocognitive disorders occurring from 1 to 12 months postoperatively.^8^ Collectively, these PNDs confer substantial morbidity and mortality among the 19 million surgical procedures performed annually in the US in adults aged 65 years or older.^11,12,13,14^

The short- and long-term consequences associated with PNDs may contribute to excessive health care costs.^13,15^ In previous studies, patients with PNDs have experienced not only increased hospital length of stay but also more frequent discharge to skilled nursing facilities.^16^ In particular, postoperative delirium has been shown to be associated with increased length of hospitalization, a 3-fold increase in the odds of discharge to a nursing facility, and a 17% increased risk of death at 1 year.^16,17^ Despite evidence supporting increased morbidity and mortality, the specific costs associated with PNDs in older adults has not been previously reported, to our knowledge.

To address these gaps, Medicare claims data were used to assess whether beneficiaries with a PND had higher health care expenditures following their index surgical procedure compared with those who did not experience a PND. Therefore, this study aimed to test the hypothesis that experiencing a PND was associated with increased inflation-adjusted total Medicare expenditures in the 1 year after surgical treatment.

## Methods

This study was approved by the Dartmouth College Committee for the Protection of Human Subjects institutional review board. Informed consent was waived owing to the retrospective nature of the study, per institutional policy. The reporting of this study was conducted in accordance with the Strengthening the Reporting of Observational Studies in Epidemiology (STROBE) reporting guideline for cohort studies.

### Assembly of the Cohort

This retrospective cohort was assembled using claims data from the Bundled Payments for Care Improvement Advanced (BPCI-A) model from January 2013 through December 2016. The BPCI-A is an initiative from the Centers for Medicare & Medicaid Services that considers the aggregate payments made from the time period that begins with the index hospitalization (the anchor episode). In this analysis, patients were eligible for inclusion if they were enrolled in Medicare fee-for-service and underwent an inpatient hospital admission associated with a surgical procedure. Patients were excluded if they were dually eligible and enrolled in both Medicare and Medicaid, younger than 65 years, or were enrolled in the Medicare End Stage Renal Disease program prior to the surgical procedure. Patients who presented with pre-existing neurocognitive diagnoses, defined based on the *International Classification of Diseases, Ninth Revision*^18^ or *Tenth Revision,*^19^ (*ICD-9/ICD-10*) codes in the year preceding the index surgical procedure, were excluded from analysis (eTable 1 in the Supplement). These conditions included dementia, cognitive impairment, altered mental status, and cognitive deficits as a result of prior stroke or traumatic brain injury. Index surgical procedures were identified using the BPCI-A methods, which include the Medicare Severity-Diagnosis Related Group codes (eTable 2 in the Supplement). The BPCI-A episodes were later bundled into 3 surgical categories, cardiac, general, and orthopedic, to streamline the presentation and interpretation of the results.

### Definition of PNDs

Several disorders associated with delayed or impaired cognitive recovery after surgical treatment were included under the definition of PND, including postoperative delirium, delayed neurocognitive recovery, and PNDs that can be classified as mild or major neurocognitive disorders occurring from 1 to 12 months postoperatively.^8^ In this study, PNDs, our primary exposure, were defined as an *ICD-9* or *ICD-10* diagnosis of delirium, mild cognitive impairment, or dementia within 1 year of discharge from the index surgical procedure (eTable 3 in the Supplement). These diagnoses were based on the *DSM-5* criteria for neurocognitive disorders and are consistent with recent recommendations for a revised PND nomenclature.^8^

### Healthcare Expenditures

The primary outcome was total inflation-adjusted Medicare postacute care payments within 1 year following the index surgical procedure. Postacute care payments represented the costs associated with readmissions, skilled nursing facilities, home health care, long-term care, physician office visits, and radiological or laboratory tests. Costs were adjusted for inflation using the Consumer Price Index and are reported in 2016 dollars.^20^

### Statistical Analysis

Descriptive statistics of the data were assessed and reported. Categorical data are presented as frequencies and proportions and assessed with a χ^2^ test. Continuous data were reported as means and SDs or medians and interquartile ranges (IQRs), depending on the distribution of the data. Normality of continuous variables was assessed with a Shapiro-Wilk test. A complete case approach was used to address missing data in the primary analysis. No imputation for missing data was performed.

Mixed-effects linear hierarchal models were used to examine the association of PND with Medicare payments while adjusting for a priori defined patient and hospital characteristics. Patient and hospital characteristics were based on their biological plausibility and significance (*P* < .10) in unadjusted analyses. A model was first fit using full maximum likelihood estimation with no predictors to evaluate the variation in payments that could be attributed to the index admission hospital. From this model, the intraclass correlation coefficient is reported. Next, a model was fit using PNDs to estimate postacute care payments, in which the hospital was included as a random effect. A similar model was fit adjusting for the prespecified patient characteristics. Lastly, a multivariable model was fit in which the association of PND with payments was assessed, including hospital as a random effect and adjusting for patient and hospital characteristics (eAppendix in the Supplement). The final model adjusted for patient characteristics, including age, sex, surgical bundle (ie, cardiac, general surgery, or orthopedic), Area Deprivation Index, community Hierarchal Condition Category score, and discharged home status. Hospital characteristics included in the final model included region of the country, designation as rural or urban, ownership (ie, government, private for-profit, private nonprofit, or other), academic medical center designation, and number of beds. Results of the final model are presented as our primary analysis and are reported as β estimates and their associated 95% CIs.

All analyses were performed using SAS statistical software version 9.4 (SAS Institute) and R statistical software version 3.6 (R Project for Statistical Computing) with 2-sided *P* < .05 considered statistically significant. Because no adjustments were performed for multiple testing, prespecified secondary outcomes should be considered exploratory and interpreted with caution. Given the observational nature of this study using retrospective data, no a priori power calculation was performed. Statistical analysis was performed between October 2019 and May 2020.

## Results

**Figure 1.**
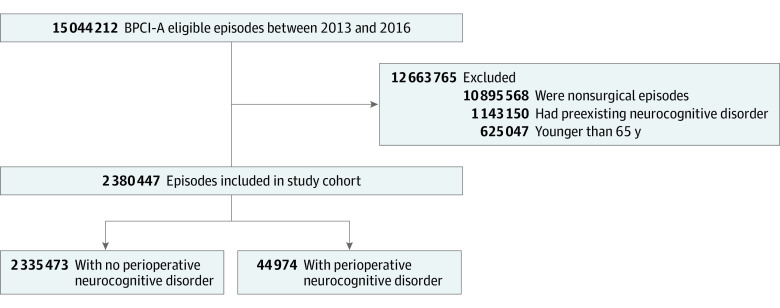
Assembly of the Cohort Data from the Bundled Payment for Care Improvement-Advanced (BPCI-A) data set were used to assess patients presenting for surgery between 2013 and 2016.

A total of 2 380 447 Medicare patients mean [SD] age, 75.36 (7.31) years; 1 336 736 [56.1%] women) who underwent surgical procedures were included, among whom 44 974 patients (1.9%) were diagnosed with a PND (Figure 1). Among all patients, most were White (2 142 157 patients [90.0%]), presenting for orthopedic surgery (1 523 782 patients [64.0%]) in urban medical centers (2 179 893 patients [91.6%]) that were private nonprofits (1 798 749 patients [75.6%]).

### Baseline Comparison of Patents With and Without a PND

Patients with a PND, compared with those without a PND, were more likely to be older (mean [SD] age, 80.65 [8.18] years vs 75.26 [7.29] years; *P* < .001), women (26 523 [59.0%] women vs 1 310 213 [56.1%] women; *P* < .001), from the northeast (11 503 patients [25.6%] vs 581 055 patients [24.9%]; *P* < .001), have more baseline comorbidities (mean [SD] Hierarchal Condition Category community score, 1.69 [1.19] vs 1.17 [0.97]; *P* < .001), and have a higher mean (SD) area deprivation index (46.60 [25.23] vs 44.31 [24.80]; *P* < .001) (Table 1).

**Table 1.** Patient and Hospital Characteristics Stratified by Postoperative Neurocognitive Status

Characteristic	Patients, No (%)		*P* Value
	No PND (n = 2 335 473)	PND (n = 44 974)	
Patient level			
Age, mean (SD), y	75.26 (7.29)	80.65 (8.18)	<.001
Women	1 310 213 (56.1)	26 523 (59.0)	<.001
Race/ethnicity			
White	2 101 502 (90.0)	40 655 (90.4)	<.001
Black	119 988 (5.1)	2485 (5.5)	
Asian	26 922 (1.2)	425 (0.9)	
North American Native	11 400 (0.5)	313 (0.7)	
Hispanic	22 901 (1.0)	508 (1.2)	
Other	28 244 (1.2)	414 (0.9)	
Unknown	24 516 (1.1)	174 (0.4)	
HCC score, mean (SD), community	1.17 (0.97)	1.69 (1.19)	<.001
Neurocognitive inpatient diagnosis	0	696 (1.5)	<.001
Area deprivation index, mean (SD)	44.31(24.80)	46.60 (25.35)	<.001
Bundle category			
Orthopedics	1 496 142 (64.1)	27 640 (61.5)	<.001
Cardiac	650 458 (27.9)	13 751 (30.6)	
General surgery	188 873 (8.1)	3583 (8.0)	
Admission year			
2013	583 884 (25.0)	5956 (13.2)	<.001
2014	556 705 (23.9)	6146 (13.7)	
2015	569 975 (24.4)	13 498 (30.0)	
2016	624 909 (26.8)	19 374 (43.1)	
Hospital level			
Region			
Midwest	418 214 (17.9)	8326 (18.5)	<.001
Northeast	581 055 (24.9)	11 503 (25.6)	
South	767 255 (32.9)	14 854 (33.0)	
West	558 535 (23.9)	10 121 (22.5)	
United States territories	10 414 (0.5)	170 (0.4)	
Community type			
Rural	195 819 (8.4)	4735 (10.5)	<.001
Urban	2 139 654 (91.6)	40 239 (89.5)	
Academic medical center	113 992 (4.9)	2281 (5.1)	.06
Bed size, mean (SD)	421.35 (374.2)	423.25 (367.1)	.29
Ownership			
Government	205 149 (8.8)	4344 (9.7)	<.001
Private for profit	365 260 (15.6)	6941 (15.4)	
Private not for profit	1 765 060 (75.6)	33 689 (74.9)	

Abbreviations: HCC, Hierarchical Condition Category; PND, postoperative neurocognitive disorder.

### Unadjusted Hospital and Payment Outcomes

Table 2 reports unadjusted outcomes stratified by PND status. Patients with a PND, compared with those without a PND, had longer acute inpatient hospital stays (mean [SD], 5.91 [6.01] days vs 4.29 [4.18] days; *P* < .001), were less likely to be discharged to home (9947 patients [22.1%] vs 914 925 [39.2%]; *P* < .001), and had a higher incidence of mortality at 1-year after index surgical treatment (4580 patients [10.2%] vs 103 767 [4.4%]; *P* < .001). Among patients who developed a PND, most experienced postoperative delirium (38 128 patients [84.8%]) followed by dementia (19 459 patients [43.3%]) and mild cognitive impairment (7999 patients [17.8%]).

**Table 2.** Patient Outcomes and Payment Characteristics

Characteristic	Patients, No. (%)		*P* value
	No PND (n = 2 335 473)	With PND (n = 44 974)	
Index hospitalization outcomes			
Discharged home	914 925 (39.2)	9947 (22.1)	<.001
Hospital length of stay, mean (SD), d	4.29 (4.18)	5.91 (6.01)	<.001
Mortality at 1 y	103 767 (4.4)	4580 (10.2)	<.001
Time to death, mean (SD), d	66.49 (79.98)	123.61 (84.64)	<.001
PND diagnoses^a^			
Dementia	NA	19 459 (43.3)	<.001
Delirium	NA	38 128 (84.8)	<.001
Mild cognitive impairment	NA	7999 (17.8)	<.001
Episode payments			
Episode payments, $	26 587.18 (17 445.65-43 448.52)	50 631.71 (31 235.87-81 890.37)	<.001
Inpatient acute payments, $	14 985.76 (11 838.94-23 530.12)	17 484.16 (12 297.97-30 485.68)	<.001
Total postacute care payments			
Postacute care payments, $	7149.35 (3053.55-19 167.01)	26 881.74 (10 544.44-54 497.84)	<.001
Inpatient payments, mean (SD), $	11 451.16 (12 392.01)	14 049.48 (13 746.48)	<.001
Readmission payments, $	13 258.03 (8089.49-23 068.84)	16 380.61 (8933.60-32 506.53)	<.001
Long-term care payments, mean (SD), $	39 101.31 (29 270.33)	40 752.25 (29 510.67)	.04
Skilled nursing facility payments, $	10 453.28 (6085.24-17 566.25)	16 819.52 (9677.44-27 943.74)	<.001
Home health payments, $	3219.71 (2523.15-4371.75)	3846.94 (2717.41-6000.17)	<.001
Hospice payments, $	3149.89 (1378.83-8148.33)	3418.10 (1457.38-7910.59)	.42
Durable medical equipment, $	181.13 (69.22-528.39)	179.39 (66.07-561.48)	.33
Outpatient payments, $	878.60 (266.67-2217.12)	1317.32 (422.31-3290.41)	<.001
Part B payments, $	1736.34 (707.10-3616.89)	3333.00 (1394.89-6726.03)	<.001

Abbreviations: NA, not applicable; PND, postoperative neurocognitive disorder.

^a^ Nonexclusive categories.

In unadjusted analyses, total inflation-adjusted 1-year payments after the initial visit were significantly higher among patients who experienced a PND (median [IQR], $26 881.74 [$10 554.44-$54 497.84]) compared with those who did not ($7149.35 [$3053.55-$19 167.01]; *P* < .001). This was especially pronounced in postacute skilled nursing facility payments for patients with PND vs those without PND (median [IQR] $16 819.52 [$9677.44-$27 943.74] vs $10 453.28 [$6085.24-$17 566.25]; *P* < .001). Episode and acute care payments for the inpatient stay tied to their index surgical admission were similarly associated with an increase. That is, total episode payments for the index inpatient hospital visit were significantly higher among those who experienced a PND compared with those who did not (median [IQR], $50 631.71 [$31 235.87-$81 890.37] vs $26 587.18 [$17 445.65-$43 448.52]; *P* < .001).

### Subtypes of PNDs and Unadjusted Payments

To assess the costs associated with each disorder, patients were stratified into subtypes of PND. Given that these were not necessarily exclusive categories, some patients experienced them in combination. Of patients with a PND, a total of 6846 patients (15.2%) experienced mild cognitive impairment in isolation and 18 221 patients (40.5%) experienced delirium alone. Mild cognitive impairment and delirium occurred in combination in 448 patients (1.0%), delirium and dementia together occurred in 18 754 patients (41.7%), and 705 patients (1.6%) experienced all 3 PND subtypes.

### Primary Analysis of Postacute Care Payments

**Figure 2.**
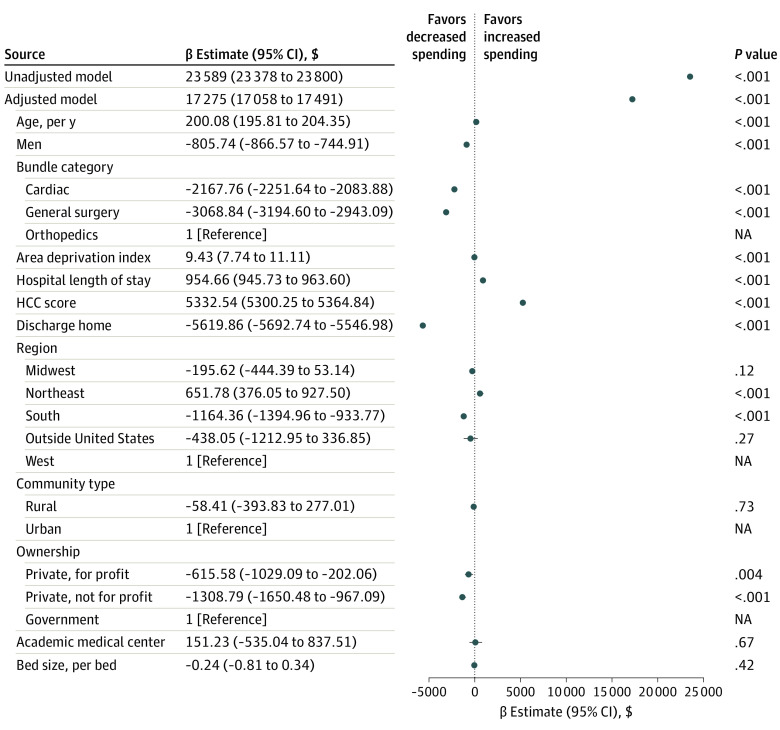
Unadjusted and Multivariable Models Predicting Health Care Costs HCC indicates Hierarchical Condition Category.

**Figure 3.**
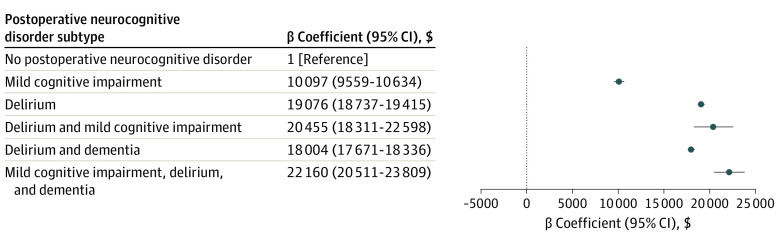
Adjusted Payments by Neurocognitive Disorder Subtype No patients were categorized as having both dementia and mild cognitive impairment in the first year following the index surgical admission and are thus not reported.

In our primary analysis, after adjusting for patient and hospital characteristics (Figure 2), presence of a PND within 1 year from the index procedure was associated with an increase in postacute care payments of $17 275 (95% CI, $17 058-$17 491) in the 1-year postadmission period (*P* < .001). In the unadjusted model, the intraclass correlation coefficient was 0.05, indicating that 5.0% of the variability in payment was attributed to the hospital. The adjusted total postacute care costs are illustrated in Figure 3. Patients with all PND subtypes had the highest postacute care payments ($22 160; 95% CI, $20 511-$23 809) followed by those with delirium and neurocognitive impairment ($20 455; 95% CI, $18 311-$22 598).

## Discussion

This cohort study found that the diagnosis of PND was associated with a significant increase in annual Medicare payments. This association was driven largely by differences in skilled nursing care costs during the 1 year following the index surgical procedure. Payments made during the index hospital admission for patients with PND were higher than for those without PND.

It is important to note that our study was not designed to establish a causal link between surgical treatment and PND. While the association of the perioperative encounter with PND has been well described, the causal links, and therefore potentially modifiable targets, remain unclear. This is reflected in the relative paucity of successful preventative or therapeutic interventions available. Our study highlights the potential cost savings if we are able to identify at-risk surgical subgroups and use targeted measures to prevent or reduce the incidence, severity, or duration of PND.^21^

In this cohort study, the presence of a PND was associated with older age, lower socioeconomic status, and a higher comorbidity burden at baseline. These findings are consistent with previous studies that have demonstrated increasing risk of PND, particularly delirium, with advancing age.^5^ In this study, PND was defined within 12 months of surgical treatment based on the most recent guidelines,^9^ which now refer to both delirium and postoperative cognitive dysfunction as PNDs and define any neurocognitive disorder present up to 12 months after surgical treatment as postoperative in etiological origin. However, there must be a recognition of longitudinal decline and an acknowledgment that sustained PND later may not be entirely attributed to the surgical treatment itself.

Our findings are consistent with a large cohort study that included 841 hospitalized patients 70 years and older enrolled in a delirium prevention trial, and found that those developing delirium had mean costs more than 2.5-fold higher.^22^ Moreover, in that study, the 1-year costs observed for an individual episode of delirium contributed an additional cost ranging from $16 000 to $64 000 (in 2005 dollars).^22^ In our study, the adjusted 1-year payment associated with postoperative delirium alone was $19 076. In addition, we found that a diagnosis of mild cognitive impairment was associated with a significant increase in the median payment, and the highest payments were observed in patients with dual diagnoses.

Prior research has shown conflicting results with regard to the association of PNDs with an increased length of inpatient hospitalizations.^23,24,25^ In this study, presence of a PND was associated with a more than 1-day increase in the mean length of stay. Although it is possible that the length of stay was associated with the increased costs for the acute in-hospital payments for the index hospitalization that was observed, it is noteworthy that the difference, albeit statistically significant, was not substantially different clinically. This finding perhaps suggests that payments made for other complications incurred during the index hospitalization (eg, urinary tract infections) were not a primary driver of the cost difference between groups.

Although exploration of cost containment in patients with PND is necessary, of importance is the preoperative optimization of surgical candidates at higher risk of complications, such as PND, as well as a global assessment of appropriateness of surgical candidacy itself.^26^ Traditional approaches to clinical risk assessment use age as a factor for increased risk of stress. Relatively recent advancements in the study of aging have led to the concept of the frailty syndrome, a state of depleted physiologic reserve and clinical risk that is associated with, but variably present with, advancing age.^17^ Further validation of initiatives around frailty assessment to ascertain readiness for surgical treatment or guide perioperative treatment are ongoing.^27^ Presumably, mitigating the incidence and extent of PND through these initiatives in individuals deemed to be at higher risk may reduce expenditure and merits consideration.

Previous studies have examined a wide variety of preventative and treatment strategies to avoid or mitigate PND, with varying success,^28^ suggesting that PND is potentially modifiable and thus hypothetically cost saving.^24^ For instance, nonpharmacological interventions, such as the pivotal multicomponent Hospital Elder Life Program, have been shown to be effective in reducing the risk of developing delirium in hospitalized older patients by 40% to 60% compared with usual care,^24,29^ which translates to an annual savings of $9446 as a result of decreased long-term nursing home expenses.^30^ In a 2011 single-center study,^31^ a delirium prevention program was estimated to yield more than $1 million in cost savings annually. Fewer studies have reported on the economic consequences associated with PNDs other than delirium. Our findings provide robust evidence that helps to bridge this gap, in which we observe an association of increased postacute care costs in patients with other forms of PND.

### Limitations

This study has some limitations, such as the use of administrative data to identify patients with a PND. Previous *ICD* validation studies for delirium and dementia have demonstrated low sensitivity and high specificity, which may explain why this cohort experienced much lower rates of PNDs than reported in prior research.^32,33,34^ However, if these are misclassified, these characteristics would likely underestimate the economic burden associated with PNDs, as false-negatives (ie, the patient had PND but was tagged as no PND) would be included in the no PND group, thus shrinking the effect size. Second, it is important to note that different modeling strategies could result in different absolute values of cost β estimates. We selected a linear model for analyzing costs because of the ease of interpreting the results. It is possible that this may have led to a model that did not have the most optimized fit for some variables. Other fields, particularly health economics, have identified other strategies to model cost data; however, the results of these models can often be hard to interpret. Thus, while it is possible that the alternative models could yield a smaller mean difference, it is unlikely that these strategies would change the conclusions we observed, namely that PNDs were associated with an increase in costs. Third, *ICD* coding transitioned from *ICD-9* to *ICD-10* during the study period. It is possible that this change resulted in miscoding, which would introduce misclassification bias, although studies suggest that the change to *ICD-10* resulted in similar coding accuracy.^33^ Fourth, post-hoc payment adjustments used by the BPCI-A could influence our findings by removing payments for certain conditions, such as for trauma and cancer. For example, if a patient in our cohort was readmitted to the hospital with a femur fracture as a result of a fall, these payments would not be reflected in our study. Fifth, this study only examined Medicare payments to estimate the economic burden and is thus unable to evaluate or comment on other payment sources. A 2016 study by Kelley^35^ suggested that delirium may be associated with a significant out-of-pocket expenditure. Sixth, our study excluded patients with preexisting neurocognitive deficits; however, preexisting cognitive impairment is a strong risk factor for PND.^28^ Further studies are needed to both identify and quantify costs in this at-risk cohort. Lastly, in contrast to delirium, which can be diagnosed using a well-validated clinical assessment tools such as the Confusion Assessment Method, the diagnosis of other forms of PNDs using *DSM-5* criteria^10^ have not been well studied.

## Conclusion

The findings of our cohort study demonstrate that there were significant Medicare costs associated with PNDs occurring in the year following surgical treatment. As PNDs are often considered the most common postoperative complication in older patients, focusing on efforts to prevent or reduce duration of symptoms may result in significant cost savings.^1,36^ Future research is needed to determine whether measures to decrease the risk of PND are associated with cost savings.

## Notes

Supplement. **eTable 1.** Pre-existing Neurocognitive Diagnoses Definitions **eTable 2.** Bundled Payments for Care Improvement Advanced Model Year 1-Episode Definitions **eTable 3.** *International Classification of Diseases*, *Ninth* or *Tenth Revision*, Diagnosis Codes for Perioperative Neurocognitive Disorders **eAppendix.** Statistical Methods
