# Supplementary material for: Economic Burden of Postoperative Neurocognitive Disorders Among US Medicare Patients
Source: JAMA Netw Open. 2020 Jul 31;3(7):e208931. doi: 10.1001/jamanetworkopen.2020.8931 (PMC7395237; doi:10.1001/jamanetworkopen.2020.8931)
Supplement: Supplement. — eTable 1. Pre-existing Neurocognitive Diagnoses Definitions eTable 2. Bundled Payments for Care Improvement Advanced Model Year 1-Episode Definitions eTable 3. International Classification of Diseases, Ninth or Tenth Revision, Diagnosis Codes for Perioperative Neurocognitive Disorders eAppendix. Statistical Methods [file jamanetwopen-3-e208931-s001.pdf]

Supplementary Online Content

Boone MD, Sites B, von Recklinghausen FM, Mueller A, Taenzer AH, Shaefi S. Economic Burden of Postoperative Neurocognitive Disorders Among US Medicare Patients. *JAMA Netw Open.* 2020;3(7):e208931. doi:10.1001/jamanetworkopen.2020.8931

eTable 1. Pre-existing Neurocognitive Diagnoses Definitions

eTable 2. Bundled Payments for Care Improvement Advanced Model Year 1-Episode Definitions

eTable 3. *International Classification of Diseases, Ninth* or *Tenth Revision,* Diagnosis Codes for Perioperative Neurocognitive Disorders

eAppendix. Statistical Methods

This supplementary material has been provided by the authors to give readers additional information about their work.

© 2020 Boone MD et al. *JAMA Network Open.*

eTable 1. Pre-existing Neurocognitive Diagnoses Definitions

Pre-existing neurocognitive diagnoses defined based on the International Classification of Diseases Ninth or Tenth Revision (ICD 9/10) Codes

Type	Description	Code(s)
ICD-10	Cognitive impairment	G31.84
ICD-10	Age related cognitive decline	R41.81
ICD-10	Altered mental status	R41.82
ICD-10	Cerebral degeneration	G31.9
ICD-10	Unspecified symptoms and signs involving cognitive functions and awareness	R41.9
ICD-10	Cognitive deficits following (sequelae of) cerebral hemorrhage or infarction	I69.01-, I69.11-, I69.21-, I69.31-, I69.81-, I69.91-
ICD-10	Cognitive impairment due to intracranial or head injury	S06.-
ICD-10	Dementia	F01.-, F02.-, F03
ICD-10	Mild memory disturbance	F06.8
ICD-10	Neurologic neglect syndrome	R41.4
ICD-10	Personality change, nonpsychotic	F68.8
ICD-9	Cognitive impairment	331.83
ICD-9	Altered mental status	780.97
ICD-9	Memory loss	780.93
ICD-9	Cerebral degeneration	331.0-331.9
ICD-9	Change in mental status	780.97
ICD-9	Cognitive deficits following (late effects of) cerebral hemorrhage or infarction	438.0
ICD-9	Cognitive impairment due to intracranial or head injury	850-854, 959.01
ICD-9	Cognitive impairment due to late effect of intracranial injury	907.0
ICD-9	Cognitive impairment due to skull fracture	800-801, 803-804
ICD-9	Dementia	290.0-290.43, 294.20-294.21
ICD-9	Unspecified persistent mental disorders due to conditions classified elsewhere	294.9
ICD-9	Mild memory disturbance	310.89
ICD-9	Neurologic neglect syndrome	781.8
ICD-9	Personality change, nonpsychotic	310.1
ICD-9	Dementia	046.1, 046.3, 290.x, 291.2, 294.1, 294.11, 294.8, 331.x (*except 331.83*), 332.0, 333.0, 333.4, 797
ICD-9	Cognitive signs and symptoms	799.5x

© 2020 Boone MD et al. *JAMA Network Open.*

eTable2. Bundled Payments for Care Improvement Advanced Model Year 1-Episode Definitions
Inpatient Clinical Episodes: Medicare Severity Diagnosis Related Groups (MS-DRG) Trigger Codes effective per the 2018 Inpatient Prospective Payment System (IPPS) Final Rule

Clinical Group	Clinical Episode	DRG # 1	DRG # 2	DRG # 3	DRG # 4	DRG # 5	DRG # 6	DRG # 7	DRG # 8
Orthopedic	Back and neck except spinal fusion	518	519	520					
Cardiac	Coronary artery bypass graft surgery	231	232	233	234	235	236		
Cardiac	Cardiac defibrillator	222	223	224	225	226	227		
Cardiac	Cardiac valve	216	217	218	219	220	221	266	267
Orthopedic	Cervical spinal fusion	471	472	473					
Orthopedic	Combined anterior posterior spinal fusion	453	454	455					
Orthopedic	Double joint replacement of the lower extremity	461	462						
Orthopedic	Fractures femur and hip/pelvis	533	534	535	536				
Orthopedic	Hip and femur procedures except major joint	480	481	482					
Orthopedic	Lower extremity and humerus procedure except hip, foot, femur	492	493	494					
General Surgery	Major bowel procedure	329	330	331					
Orthopedic	Major joint replacement of the lower extremity	469	470						
Orthopedic	Major joint replacement of upper extremity	483							
Cardiac	Pacemaker	242	243	244					
Cardiac	Percutaneous coronary intervention	246	247	248	249	250	251	273	274
Orthopedic	Spinal fusion (non-cervical)	459	460						

© 2020 Boone MD et al. *JAMA Network Open.*

eTable 3. *International Classification of Diseases, Ninth* or *Tenth Revision*, Diagnosis Codes for Perioperative Neurocognitive Disorders

Perioperative Neurocognitive Disorder	ICD 9 Diagnosis	ICD 10 Diagnosis
Delirium	'29011', '2903', '29041', '2910', '2930', '2931', '2900', '29389', '2899'	'R410', 'F0390', 'F0391'
Mild Cognitive Impairment	'33183'	'G3184'
Dementia	'29011', '2903', '29041', '2900'	'F0390', 'F0391'
Abbreviations: ICD = International Classification of Diseases		

© 2020 Boone MD et al. *JAMA Network Open.*

eAppendix. Statistical Methods

We used linear mixed models to characterize Medicare post acute payments within and between hospitals. Level one variables were patient predictors including presence of an neurocognitive disorder, age in years, gender, length of stay, Hierarchical Condition Category (HCC) score, area deprivation index, surgical bundle and discharge to home. Level 2 included hospital predictors included region of the country, rural or urban, ownership, academic medical center and the number of beds. All models accounted for the clustering of patients within hospitals using a random effect. We characterized the hospitals' effect by using the variance decomposition from the random effects term in a null model (Model1) with Medicare post visit payments entered as the outcome variable.

**Model 1<br>Intercept Only**		
**Effect**	**β Estimate<br>(95% Confidence Interval)**	**P Value**
Intercept	$17036.00 (16850.00,17222.00)	<.0001

In Model 2, only PND was included as a fixed effect, including the clustering of hospital as a random effect.

**Model 2<br>Intercept + PND Fixed Effect**		
**Effect**	**β Estimate<br>(95% Confidence Interval)**	**P Value**
Intercept	$16468 (16286, 16650)	<.0001
PND	$23589 (23378, 23800)	<.0001
No PND	[REFERENCE]	

In Model 3, we assessed the relationship between Level 1 characteristics in a multivariable model by setting payments as the outcome specifying fixed linear effects for the patient (Level 1).

**Model 3<br>Patient Fixed Effects**		
**Effect**	**β Estimate<br>(95% Confidence Interval)**	**P Value**
Intercept	-$5903.20 (-6267.74, -5538.65)	<.0001
PND	$17275.00 (17058.00, 17492.00)	<.0001
No PND	[REFERENCE]	
Age, *years*	$200.22 (195.95, 204.48)	<.0001
Male	-$807.60 (-868.43, -746.76)	<.0001
Female	[REFERENCE]	

© 2020 Boone MD et al. *JAMA Network Open.*

Length of Stay	$955.02 (946.08, 963.96)	<.0001
Area Deprivation Index	$8.28 (6.62, 9.95)	<.0001
Cardiac bundles	-$2166.79 (-2250.61, -2082.97)	<.0001
General Surgery Bundles	-$3071.29 (-3197.06, -2945.53)	<.0001
Orthopedics Bundles	[REFERENCE]	
HCC Score	$5332.17 (5299.88, 5364.46)	<.0001
Discharge Home	-$5621.54 (-5694.42, -5548.66)	<.0001
Discharge not Home	[REFERENCE]	

**Model 3<br>Model Fit Characteristics**	
AIC	45478337
BIC	45478413

Finally, in Model 4 we assessed the relationship between the payments and both the fixed patient (Level 1) and hospital (Level 2) characteristics using multivariable models specifying linear effects for all characteristics. Similarly, this model accounted for the clustering of patients within hospitals using a random effect. Model improvement was assessed using the Akaike's Information Criterion (AIC) and Bayesian Information Criterion (BIC) when the nested models differ in fixed effects. For both of these measures, smaller values represent better fitting models. The results of Model 4 are presented in the manuscript as our primary analysis.

Model 4
Patient and Hospital Effects

**Effect**	**β Estimate (95% Confidence Interval)**	**P Value**
Intercept	-$4601.06 (-5125.35, -4076.76)	<.0001
PND	$17275.00 (17058.00, 17491.00)	<.0001
No PND	[REFERENCE]	
Age, *years*	$200.08 (195.81, 204.35)	<.0001
Male	-$805.74 (-866.57, -744.91)	<.0001
Female	[REFERENCE]	
Length of Stay	$954.66 (945.73, 963.60)	<.0001
Area Deprivation Index	$9.43 (7.74, 11.11)	<.0001
Cardiac bundles	-$2167.76 (-2251.64, -2083.88)	<.0001
General Surgery Bundles	-$3068.84 (-3194.60, -2943.09)	<.0001
Orthopedics Bundles	[REFERENCE]	
HCC Score	$5332.54 (5300.25, 5364.84)	<.0001
Discharge Home	-$5619.86 (-5692.74, -5546.98)	<.0001
Discharge not Home	[REFERENCE]	

© 2020 Boone MD et al. *JAMA Network Open.*

Midwest	-$195.62 (-444.39, 53.14)	0.1232
Northeast	$651.78 (376.05, 927.50)	<.0001
Out of US	-$438.05 (-1212.95, 336.85)	0.2678
South	-$1164.36 (-1394.96, -933.77)	<.0001
West	[REFERENCE]	
Rural	-$58.41 (-393.83, 277.01)	0.7328
Urban	[REFERENCE]	
Other	-$5612.32 (-30273.00, 19048.00)	0.6554
Private for profit	-$615.58 (-1029.09, -202.06)	0.0036
Private not for profit	-$1308.79 (-1650.48, -967.09)	<.0001
Government	[REFERENCE]	
Academic Medical Center	$151.23 (-535.04, 837.51)	0.6657
Not Academic Medical Center	[REFERENCE]	
Beds	-$0.24 (-0.81, 0.34)	0.4199

**Model 4<br>Model Fit Characteristics**	
AIC	45478028
BIC	45478168

© 2020 Boone MD et al. *JAMA Network Open.*
